# Multimodal Machine Learning-Based Technical Failure Prediction in Patients Undergoing Transcatheter Aortic Valve Replacement

**DOI:** 10.1016/j.jacadv.2025.102168

**Published:** 2025-09-18

**Authors:** Daijiro Tomii, Isaac Shiri, Giovanni Baj, Masaaki Nakase, Pooya Mohammadi Kazaj, Daryoush Samim, Joanna Bartkowiak, Fabien Praz, Jonas Lanz, Stefan Stortecky, David Reineke, Stephan Windecker, Thomas Pilgrim, Christoph Gräni

**Affiliations:** a Department of Cardiology, Inselspital, University of Bern, Bern, Switzerland; b Department of Cardiac Surgery, Inselspital, University of Bern, Bern, Switzerland

**Keywords:** aortic stenosis, machine learning, transcatheter aortic valve replacement, valve Academic research Consortium

## Abstract

**Background:**

Technical failure is not uncommon and is associated with unfavorable outcomes in patients undergoing TAVR. However, predicting procedural failure remains challenging due to the complex interplay of clinical, anatomical, and procedural factors.

**Objectives:**

The objective of the study was to develop and validate a data-driven prediction model for technical failure of transcatheter aortic valve replacement (TAVR), using multimodal information and machine learning algorithms.

**Methods:**

In a prospective TAVR registry, 184 parameters derived from clinical examination, laboratory studies, electrocardiography, echocardiography, cardiac catheterization, computed tomography, and procedural measurements were used for machine learning modeling of TAVR technical failure prediction. For the machine learning algorithm, 24 different model combinations were developed using a standardized machine learning pipeline. All model development steps were performed solely on the training set, whereas the holdout test set was kept separate for final evaluation. Technical success/failure was defined according to the Valve Academic Research Consortium (VARC)-3 definition, which differentiates between vascular and cardiac complications.

**Results:**

Among 2,937 consecutive patients undergoing TAVR, the rate of cardiac and vascular technical failure was 2.4% and 7.0%, respectively. For both categories of technical failure, the best-performing model demonstrated moderate-to-high discrimination (cardiac: area under the curve: 0.769; vascular: area under the curve: 0.788), with high negative predictive values (0.995 and 0.976, respectively). Interpretability analysis showed that atherosclerotic comorbidities, computed tomography-based aortic root and iliofemoral anatomies, antithrombotic management, and procedural features were consistently identified as key determinants of VARC-3 technical failure across all models.

**Conclusions:**

Machine learning-based models that integrate multimodal data can effectively predict VARC-3 technical failure in TAVR, refining patient selection and optimizing procedural strategies.

Transcatheter aortic valve replacement (TAVR) has been shown to be noninferior or superior to surgical aortic valve replacement in a series of randomized clinical trials, leading to Class I A indications in patients with severe, symptomatic aortic stenosis across the spectrum of surgical risk.^1^^,^^2^ Procedural success is paramount to achieving optimal clinical outcomes with TAVR. Owing to improved patient selection, computed tomography (CT)–guided imaging analysis of the aortic root and access vessels, refined implantation techniques, increased operator experience, and advancements in TAVR technology, the rate of procedural complications has declined; however, a non-negligible proportion of patients remain at risk for procedural failure in contemporary TAVR practice.^3^, ^4^, ^5^ Since procedural failure is associated with unfavorable clinical outcomes,^4^^,^^5^ identification of cases at high-risk of procedural failure is crucial. However, accurately predicting procedural failure remains challenging due to the complex interplay of patient-specific, anatomical, and procedural factors.^6^, ^7^, ^8^, ^9^ The integration of multimodal data, including clinical, imaging, and procedural characteristics, may improve predictive accuracy but remains underexplored.

Machine learning algorithms have been applied in cardiovascular medicine, including TAVR, to improve risk stratification and outcome prediction.^10^, ^11^, ^12^ By integrating multimodal data from clinical examination, laboratory studies, electrocardiography, echocardiography, cardiac catheterization, CT, and procedural measurements, machine learning-based prediction models for procedural failure offer the potential to improve patient selection and optimize treatment strategies. In this study, we aimed to develop a data-driven prediction model for procedural failure in TAVR, using multimodal information and machine learning algorithms.

## Methods

### Study design and population

All patients undergoing TAVR at the Bern University Hospital, Bern, Switzerland, are consecutively recorded in a prospective institutional database as part of the SwissTAVI Registry (NCT01368250), which is mandated by Swiss health authorities.^13^ The present study screened all patients who underwent TAVR between August 2007 and June 2023. However, only those treated with contemporary-generation devices, that is, balloon-expandable (SAPIEN 3/SAPIEN 3 Ultra [Edwards Lifesciences]) or self-expanding (Evolut R/PRO/PRO Plus [Medtronic], Portico/Navitor [Abbott], or ACURATE neo/neo2 [Boston Scientific]), were included in the final analysis. Patients treated with earlier-generation devices (SAPIEN THV/XT [Edwards Lifesciences], CoreValve [Medtronic], and Lotus/Lotus Edge [Boston Scientific]) or those undergoing TAVR for pure aortic regurgitation were excluded. The registry is approved by the Bern Cantonal Ethics Committee, and patients provided written informed consent to participate. The study adheres to the Transparent Reporting of a Multivariable Prediction Model for Individual Prognosis or Diagnosis + Artificial Intelligence statement.^14^

### TAVR procedure

TAVR was performed as previously described.^4^^,^^7^ Transfemoral access was the default strategy, with alternative access routes (transaxillary, transcarotid, transcaval, and transapical) reserved for patients with unfavorable peripheral anatomy. Device selection was performed according to the anatomic and clinical criteria during a heart team discussion. For transfemoral TAVR, an ultrasound-guided transfemoral access approach has been used since 2019, and the puncture site was closed by default using the first-generation (Prostar [Abbott Vascular Inc]) and newer-generation (ProGlide/ProStyle [Abbott Vascular Inc]) suture-based devices, or a plug-based device (MANTA [Teleflex]). Surgical cut down was reserved for cases with heavily diseased femoral access in which the hemostatic devices were deemed unfeasible or for nontransfemoral access.

### Data collection and clinical endpoints

All baseline clinical, procedural, and follow-up data were prospectively recorded in a dedicated database, held at the Clinical Trials Unit of the University of Bern. Echocardiographic and CT examinations were performed as previously described, and all measurements were re-evaluated by dedicated imaging specialists and integrated into the database.^7^^,^^15^, ^16^, ^17^ Aortic valve leaflet and left ventricular outflow tract calcium volume were quantified in the contrast images by using a Hounsfield unit threshold of 850 as previously suggested.^15^^,^^18^ Iliofemoral vascular anatomy was assessed according to the Hostile score, which integrates the extent (number of lesions, lesion length, and minimum lumen diameter) and complexity (tortuosity, calcification, and the presence of obstruction) of iliofemoral atherosclerosis.^7^^,^^19^ Operator experience was quantified as the number of TAVRs performed as a primary operator in the year prior each indexed TAVR case.^20^ We adopted 2 approaches to integrate the operator experience into each machine learning algorithm: as a categorical variable (low 0-20; intermediate 21-50; intermediate-to-high 51-100; and high >100); and as a continuous variable.^20^^,^^21^ Clinical follow-up data after TAVR were obtained by standardized interviews, documentation from referring physicians, and hospital discharge summaries as previously described.^22^ All adverse events were systematically collected and adjudicated by a dedicated clinical event committee on the basis of the Valve Academic Research Consortium (VARC) criteria applicable at the time of the procedure.^23^, ^24^, ^25^ Procedural failure was defined as a technical failure, the inverse of VARC-3 technical success: 1) freedom from death; 2) successful access, delivery of the device, and retrieval of the delivery system; 3) correct positioning of a single prosthetic heart valve into the proper anatomic location; and 4) freedom from surgery or intervention related to the device or a major vascular or access-related or cardiac structural complication. Technical success/failure was retrospectively adjudicated based on detailed documentation of adjudicated endpoints that form the individual components of this composite endpoint.^4^^,^^25^ For the purpose of this analysis, technical failure was categorized into vascular technical failure, limited to vascular complications, and cardiac technical failure, including all others (Supplemental Table 1).^4^

### Data preparation

We extracted the following number of features from each modality: 79 features from clinical variables, 11 from laboratory markers, 5 from electrocardiography parameters, 36 from echocardiographic or invasive measurements, 40 from CT measurements, and 13 from procedural features (Supplemental Information).

### Model development and validation

The design and reporting of this study comply with established guidelines for artificial intelligence (AI) applications in medicine including Standards for Reporting of Diagnostic Accuracy Studies-AI,^26^ and Minimum Information for Medical AI Reporting.^27^ The relevant criteria from these guidelines have been meticulously integrated into the development, validation, and testing phases of the AI-based model.

#### Data split and feature preprocessing

The data set was initially divided into a training set (70%) and a holdout test set (30%) using stratified sampling to ensure a proportional representation of cardiac and vascular technical failure cases. Continuous variables were imputed using the mean and categorical variables were imputed using the median. To ensure consistency in scale, all continuous variables were normalized using the Z-score method. Features with low variance (threshold <0.99) were removed. In addition, highly correlated features (Pearson correlation coefficient >0.95) were clustered, retaining only the most predictive variable using univariate analysis from each group to reduce redundancy.

#### Machine learning algorithms

After the initial preprocessing, multiple feature selection algorithms, such as analysis of variance (ANOVA), Kruskal-Wallis (KW), recursive feature elimination (RFE), and Relief, were independently applied to identify informative features. Using the selected features, various machine learning classifiers were trained, including random forest (RF), Lasso logistic regression, logistic regression (LR), linear discriminant analysis, autoencoder, and AdaBoost. Altogether, for each task, twenty-four different combinations (4 feature selections and 6 classifiers) of machine learning were developed.^28^

#### Machine learning model development

All model development steps, including data imputation, feature normalization, feature selection, model parameter tuning, hyperparameter optimization, and model selection, were conducted exclusively on the training set using a 10-fold cross-validation. The holdout test set (30%) remained untouched until the final evaluation stage. Once the best model was identified for each configuration on the training set, all preprocessing steps, from data imputation to model inference (with the exact setting of the final model developed on the training set), were applied to the test set. These procedures were implemented to prevent any possibility of data leakage between the training and test sets (Central Illustration).

**Central Illustration:**
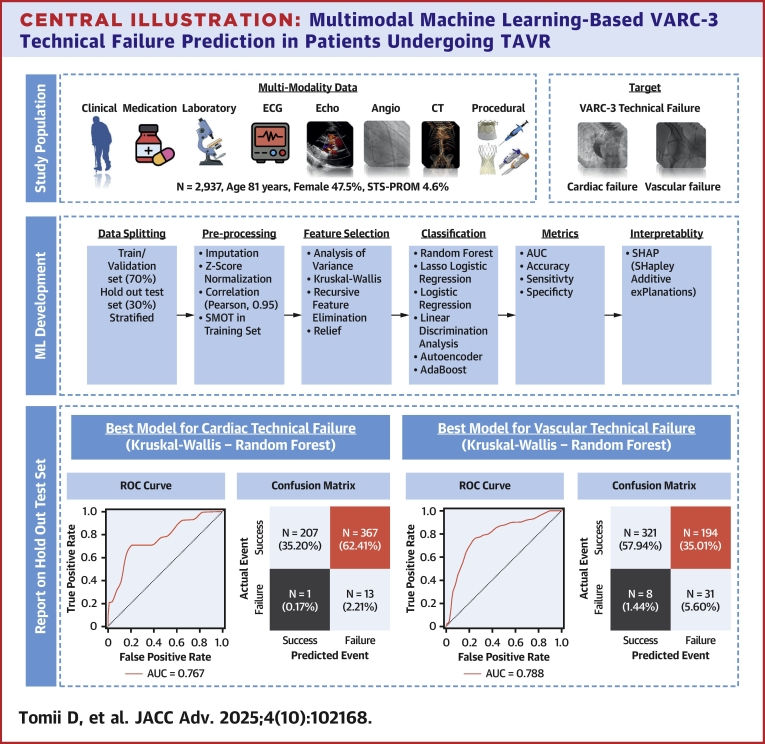
**Multimodal Machine Learning-Based VARC-3 Technical Failure Prediction in Patients Undergoing TAVR** Study population (*top*), machine-learning workflow (*middle*), and the best-performing machine-learning models for predicting VARC-3 technical failure (*bottom*). ROC curves and confusion matrixes show the best-performing models for predicting each category of technical failure: cardiac technical failure (Kruskal-Wallis – Random Forest) and vascular technical failure (Kruskal-Wallis – Random Forest). Angio = angiography; AUC = area under the curve; CT = computed tomography; ECG = electrocardiogram; Echo = echocardiography; ML = machine-learning; ROC = receiver operating characteristic; SHAP = SHapley Additive exPlanations; SMOT = Synthetic Minority Oversampling Technique; STS-PROM = Society of Thoracic Surgeons Predicted Risk of Mortality; TAVR = transcatheter aortic valve replacement; VARC = Valve Academic Research Consortium.

#### Model Performance Evaluation and Interpretability

The optimal threshold for calculating false positives, true positives, false negatives, and true negatives for further metric calculation was determined using the training set and subsequently applied to the test set. The performance of these models was evaluated using various metrics, including the area under the receiver operating characteristic curve (ROC-AUC), accuracy, sensitivity, specificity, and negative predictive value. To interpret the outputs of the machine learning models, we used the Shapley Additive Explanations (SHAP) model, which provides insights into the contribution of individual features to VARC-3 technical failure predictions.

### Statistical analysis and machine learning model evaluation

Categorical variables are presented as frequencies and percentages, whereas continuous variables are expressed as mean ± SD, or median (IQR) depending on the normality of the distribution. The area under the curve of different machine learning models was compared using the DeLong test. Statistical analyses were performed using R (version 4.4.2; R Foundation for Statistical Computing) and Python. All machine learning algorithms were implemented using Scikit-learn in Python, and all code and models are publicly available in the AI-CVM GitHub repository page (https://github.com/AI-in-Cardiovascular-Medicine).

## Results

### Study population and prevalence of VARC-3 technical failure

A total of 2,937 patients met the inclusion criteria and underwent TAVR with contemporary-generation devices between February 2014 and June 2023. All patients had sufficient data to assess technical failure according to the VARC-3 definitions and were eligible for inclusion in the present study. For the analysis of cardiac technical failure, all 2,937 patients who underwent transfemoral or nontransfemoral TAVR were included. For vascular technical failure, 2,769 patients who underwent transfemoral TAVR were available for analysis. Overall, 47.5% of the patients were female, the mean age of the cohort was 81 ± 6 years, and the Society of Thoracic Surgeons Predicted Risk of Mortality score (STS-PROM) was 4.6 ± 3.8. Among 2,937 patients, 70 patients (2.4%) developed VARC-3 cardiac technical failure (train/validation set: N = 56/2,349 [2.4%]; holdout test set: N = 14/588 [2.4%]). In the analysis of vascular technical failure, 195 of 2,769 patients (7.0%) developed VARC-3 vascular technical failure: failure (train/validation set: N = 156/2,349 [7.0%]; holdout test set: N = 39/554 [7.0%]). Key baseline clinical, echocardiographic, and CT characteristics, as well as procedural features and components of technical failure, are summarized in Table 1 and Supplemental Table 1.

**Table 1:** Clinical, Imaging, and Procedural Characteristics in Train/Validation and Holdout Test Sets

Variables	Cardiac Technical Failure			Vascular Technical Failure		
	Overall (N = 2,937)	Train/Validation Set (n = 2,349)	Holdout Test Set (n = 588)	Overall (N = 2,769)	Train/Validation Set (n = 2,215)	Holdout Test Set (n = 554)
Age, y	81 ± 6	81 ± 6	82 ± 6	81 ± 6	81 ± 6	81 ± 6
Female, n (%)	1,394 (47.5)	1,101 (46.9)	293 (49.8)	1,317 (47.6)	1,050 (47.4)	267 (48.2)
Body mass index, kg/m^2^	26.8 ± 5.4	26.8 ± 5.4	26.7 ± 5.5	26.9 ± 5.4	26.8 ± 5.4	27.2 ± 5.5
Body surface area, m^2^	1.9 ± 0.3	1.9 ± 0.3	1.9 ± 0.3	1.9 ± 0.3	1.9 ± 0.3	1.9 ± 0.3
NYHA functional class III or IV, n (%)	1,729 (58.9)	1,401 (59.7)	328 (55.8)	1,630 (58.9)	1,294 (58.4)	336 (60.6)
STS-PROM, %	4.6 ± 3.8	4.6 ± 3.8	4.7 ± 3.7	4.5 ± 3.6	4.5 ± 3.6	4.5 ± 3.8
Valve-in-valve procedure, n (%)	142 (4.8)	116 (4.9)	26 (4.4)	132 (4.8)	99 (4.5)	33 (6.0)
Urgent TAVR, n (%)	52 (1.8)	42 (1.8)	10 (1.7)	46 (1.7)	39 (1.8)	7 (1.3)
Comorbidity and medical history						
Hypertension, n (%)	2,588 (88.1)	2,065 (87.9)	523 (88.9)	2,432 (87.8)	1,939 (87.5)	493 (89.0)
Diabetes, n (%)	841 (28.6)	673 (28.7)	168 (28.6)	795 (28.7)	637 (28.8)	158 (28.5)
Dyslipidemia, n (%)	2,032 (69.2)	1,624 (69.1)	408 (69.4)	1,898 (68.5)	1,517 (68.5)	381 (68.8)
Renal failure (eGFR <60 mL/min/1.73 m^2^), n (%)	1,899 (64.7)	1,503 (64.1)	396 (67.3)	1,786 (64.6)	1,427 (64.5)	359 (64.8)
Dialysis, n (%)	66 (2.2)	56 (2.4)	10 (1.7)	61 (2.2)	47 (2.1)	14 (2.5)
Coronary artery disease, n (%)	1,622 (55.2)	1,285 (54.7)	337 (57.3)	1,500 (54.2)	1,203 (54.3)	297 (53.6)
History of myocardial infarction, n (%)	386 (13.1)	308 (13.1)	78 (13.3)	354 (12.8)	284 (12.8)	70 (12.6)
History of percutaneous coronary intervention, n (%)	772 (26.3)	601 (25.6)	171 (29.1)	717 (25.9)	585 (26.4)	132 (23.8)
History of cardiac surgery, n (%)	399 (13.6)	317 (13.5)	82 (13.9)	345 (12.5)	280 (12.6)	65 (11.7)
History of aortic valvuloplasty, n (%)	134 (4.6)	111 (4.7)	23 (3.9)	128 (4.6)	102 (4.6)	26 (4.7)
Atrial fibrillation, n (%)	986 (33.6)	805 (34.3)	181 (30.8)	915 (33.0)	751 (33.9)	164 (29.6)
Peripheral artery disease, n (%)	342 (11.6)	269 (11.5)	73 (12.4)	254 (9.2)	194 (8.8)	60 (10.8)
Carotid artery disease, n (%)	172 (6.2)	137 (6.2)	35 (6.2)	134 (5.1)	104 (5.0)	30 (5.6)
COPD, n (%)	291 (9.9)	249 (10.6)	42 (7.1)	251 (9.1)	201 (9.1)	50 (9.0)
Previous permanent pacemaker implantation, n (%)	227 (7.7)	181 (7.7)	46 (7.8)	212 (7.7)	170 (7.7)	42 (7.6)
History of cerebrovascular event, n (%)	368 (12.5)	282 (12.0)	86 (14.6)	333 (12.0)	265 (12.0)	68 (12.3)
Baseline antithrombotic therapy						
Aspirin, n (%)	1,515 (51.6)	1,205 (51.3)	310 (52.7)	1,401 (50.6)	1,096 (49.5)	305 (55.1)
P2Y12 inhibitor, n (%)	477 (16.3)	363 (15.5)	114 (19.4)	438 (15.8)	341 (15.4)	97 (17.5)
Oral anticoagulant, n (%)	965 (32.9)	762 (32.5)	203 (34.5)	905 (32.7)	735 (33.2)	170 (30.7)
Echocardiography						
Aortic valve area, cm^2^	0.92 ± 0.33	0.91 ± 0.33	0.92 ± 0.31	0.92 ± 0.33	0.92 ± 0.33	0.92 ± 0.31
Mean aortic valve gradient, mm Hg	38.6 ± 16.4	38.9 ± 16.7	37.6 ± 14.8	38.7 ± 16.4	38.8 ± 16.4	38.4 ± 16.2
Left ventricular ejection fraction, %	55.5 ± 13.2	55.4 ± 13.1	55.7 ± 13.4	55.5 ± 13.2	55.4 ± 13.3	55.9 ± 12.8
Moderate or severe aortic regurgitation, n (%)	278 (9.5)	222 (9.5)	56 (9.5)	250 (9.0)	190 (8.6)	60 (10.8)
Moderate or severe mitral regurgitation, n (%)	508 (17.3)	388 (16.5)	120 (20.4)	471 (17.0)	388 (17.5)	85 (15.3)
Moderate or severe tricuspid regurgitation, n (%)	318 (10.8)	248 (10.6)	70 (11.9)	300 (10.8)	245 (11.1)	55 (9.9)
Computed tomography						
Bicuspid valve, n (%)	114 (3.9)	92 (3.9)	22 (4.0)	108 (4.3)	87 (4.3)	21 (4.2)
Type 0	21 (0.8)	17 (0.8)	4 (0.7)	18 (0.7)	17 (0.8)	1 (0.2)
Type 1	78 (2.9)	60 (2.8)	18 (3.3)	75 (3.0)	60 (3.0)	15 (3.0)
Type 2	15 (0.6)	15 (0.7)	0 (0.0)	15 (0.6)	10 (0.5)	5 (1.0)
Aortic annulus area, mm^2^	451.5 ± 93.3	451.0 ± 93.2	453.5 ± 93.7	452.2 ± 93.6	453.0 ± 93.9	449.0 ± 92.4
Aortic annulus perimeter, mm	76.8 ± 16.4	76.8 ± 18.0	76.6 ± 7.8	76.9 ± 16.8	77.0 ± 18.4	76.3 ± 7.6
Annulus eccentricity	0.77 ± 0.07	0.77 ± 0.07	0.77 ± 0.07	0.77 ± 0.07	0.77 ± 0.07	0.77 ± 0.07
Left ventricular outflow tract area, mm^2^	424.7 ± 128.5	425.1 ± 127.6	422.8 ± 132.2	426.0 ± 129.6	427.6 ± 131.8	419.4 ± 120.4
Sinus of Valsalva diameter, mm	31.8 ± 4.5	31.8 ± 4.6	31.7 ± 4.4	31.8 ± 4.5	31.8 ± 4.6	31.7 ± 4.2
Sinotubular junction diameter, mm	29.1 ± 5.8	29.1 ± 6.3	29.0 ± 3.6	29.1 ± 6.0	29.0 ± 3.5	29.5 ± 11.3
Left coronary height, mm	15.0 ± 3.6	14.9 ± 3.6	15.1 ± 3.6	15.0 ± 3.6	15.0 ± 3.6	14.9 ± 3.6
Right coronary height, mm	18.2 ± 3.5	18.2 ± 3.5	18.2 ± 3.6	18.2 ± 3.5	18.2 ± 3.6	18.0 ± 3.4
Aortic angulation, ˚	49.7 ± 9.6	49.7 ± 9.7	49.6 ± 9.5	49.9 ± 9.6	49.8 ± 9.5	50.3 ± 9.8
Ascending aorta diameter, mm	33.3 ± 3.5	33.3 ± 3.5	33.2 ± 3.6	33.3 ± 3.5	33.3 ± 3.5	33.2 ± 3.5
Aortic valvular complex calcium volume, mm^3^	295.6 ± 287.2	296.0 ± 285.2	294.3 ± 295.3	294.5 ± 286.4	296.7 ± 288.5	285.9 ± 277.7
Left ventricular outflow tract calcium volume, mm^3^	11.4 ± 31.9	11.4 ± 30.9	11.6 ± 35.4	11.3 ± 31.2	12.1 ± 33.4	7.9 ± 20.1
Left ventricular outflow tract calcification, n (%)						
None	1,640 (65.7)	1,304 (65.7)	336 (65.9)	1,557 (66.1)	1,232 (65.5)	325 (68.7)
Mild	338 (13.5)	264 (13.3)	74 (14.5)	309 (13.1)	242 (12.9)	67 (14.2)
Moderate	241 (9.7)	194 (9.8)	47 (9.2)	229 (9.7)	188 (10.0)	41 (8.7)
Severe	276 (11.1)	223 (11.2)	53 (10.4)	260 (11.0)	220 (11.7)	40 (8.5)
Device landing zone calcium volume, mm^3^	304.9 ± 297.8	304.9 ± 294.0	304.6 ± 312.3	303.7 ± 296.6	306.2 ± 299.7	293.8 ± 283.5
Porcelain aorta, n (%)	83 (3.2)	67 (3.2)	16 (3.0)	58 (2.3)	44 (2.2)	14 (2.8)
Hostile score	1.0 [0.0, 4.0]	1.0 [0.0, 4.0]	1.0 [0.0, 3.8]	1.0 [0.0, 7.0]	1.0 [0.0, 7.0]	1.0 [0.0, 7.0]
Number of segments with significant disease, n (%)						
0	1,446 (72.2)	1,160 (71.7)	286 (74.1)	1,446 (72.2)	1,166 (72.1)	280 (72.5)
1	356 (17.8)	295 (18.2)	61 (15.8)	356 (17.8)	286 (17.7)	70 (18.1)
2	156 (7.8)	130 (8.0)	26 (6.7)	156 (7.8)	130 (8.0)	26 (6.7)
3	45 (2.2)	32 (2.0)	13 (3.4)	45 (2.2)	35 (2.2)	10 (2.6)
Presence of obstruction, n (%)	3 (0.1)	3 (0.2)	0 (0.0)	3 (0.1)	3 (0.2)	0 (0.0)
Iliac disease involving the aortic bifurcation, n (%)	1,352 (67.5)	1,102 (68.2)	250 (64.8)	1,352 (67.5)	1,097 (67.8)	255 (66.1)
≥180˚ arch calcified lesion, n (%)	329 (16.4)	261 (16.1)	68 (17.6)	329 (16.4)	265 (16.4)	64 (16.6)
Total lesion length >100 mm, n (%)	558 (27.9)	458 (28.3)	100 (25.9)	558 (27.9)	452 (28.0)	106 (27.5)
Minimal lumen diameter <5 mm, n (%)	557 (27.8)	457 (28.3)	100 (25.9)	557 (27.8)	451 (27.9)	106 (27.5)
Lesion in tortuous segment, n (%)	9 (0.4)	8 (0.5)	1 (0.3)	9 (0.4)	9 (0.6)	0 (0.0)
Procedural characteristics						
General anesthesia, n (%)	453 (15.4)	356 (15.2)	97 (16.5)	288 (10.4)	239 (10.8)	49 (8.8)
Main access site, n (%)						
Femoral	2,769 (94.3)	2,217 (94.4)	552 (93.9)	2,769 (100)	2,215 (100)	554 (100)
Transapical	134 (4.6)	108 (4.6)	26 (4.4)			
Subclavian	9 (0.3)	7 (0.3)	2 (0.3)			
Direct aortic	1 (<0.1)	1 (<0.1)	0 (0.0)			
Other	24 (0.8)	16 (0.7)	8 (1.4)			
Surgical access, n (%)	154 (5.2)	121 (5.2)	33 (5.6)	11 (0.4)	9 (0.4)	2 (0.4)
Size of predilatation balloon, mm	22.1 ± 2.5	22.1 ± 2.5	22.1 ± 2.5	22.2 ± 2.5	22.2 ± 2.6	22.1 ± 2.2
TAVR device type, n (%)						
Balloon-expandable valve	1,736 (59.2)	1,407 (60.0)	329 (56.0)	1,642 (59.4)	1,313 (59.4)	329 (59.4)
Self-expanding valve	1,195 (40.8)	937 (40.0)	258 (44.0)	1,121 (40.6)	896 (40.6)	225 (40.6)
TAVR device, n (%)						
SAPIEN 3	871 (29.7)	713 (30.4)	158 (26.9)	796 (28.8)	648 (29.3)	148 (26.7)
SAPIEN 3 Ultra	865 (29.5)	694 (29.6)	171 (29.1)	846 (30.6)	665 (30.1)	181 (32.7)
Evolut R	340 (11.6)	271 (11.6)	69 (11.8)	328 (11.9)	267 (12.1)	61 (11.0)
Evolut PRO	330 (11.3)	259 (11.0)	71 (12.1)	322 (11.7)	253 (11.5)	69 (12.5)
Evolut PRO Plus	164 (5.6)	125 (5.3)	39 (6.6)	164 (5.9)	134 (6.1)	30 (5.4)
Acurate NEO	262 (8.9)	197 (8.4)	65 (11.1)	209 (7.6)	165 (7.5)	44 (7.9)
Acurate NEO 2	18 (0.6)	13 (0.6)	5 (0.9)	18 (0.7)	13 (0.6)	5 (0.9)
Portico	33 (1.1)	30 (1.3)	3 (0.5)	33 (1.2)	27 (1.2)	6 (1.1)
Navitor	48 (1.6)	42 (1.8)	6 (1.0)	47 (1.7)	37 (1.7)	10 (1.8)
Valve size, mm	26.3 ± 2.2	26.3 ± 2.2	26.3 ± 2.3	26.4 ± 2.2	26.4 ± 2.2	26.2 ± 2.2
Sheath size, F	14.0 [14.0, 16.0]	14.0 [14.0, 16.0]	14.0 [14.0, 16.0]	14.0 [14.0, 16.0]	14.0 [14.0, 16.0]	14.0 [14.0, 16.0]
Vascular closure device used, n (%)	2,745 (93.5)	2,199 (93.6)	546 (92.9)	2,727 (98.5)	2,186 (98.7)	541 (97.7)
Type of vascular closure device, n (%)						
PerClose/ProGlide	2,172 (84.7)	1,732 (84.4)	440 (85.6)	2,162 (84.7)	1,724 (84.3)	438 (86.2)
Prostar	309 (12.0)	250 (12.2)	59 (11.5)	308 (12.1)	251 (12.3)	57 (11.2)
MANTA	84 (3.3)	69 (3.4)	15 (2.9)	84 (3.3)	71 (3.5)	13 (2.6)
Physician volume for primary operator, median case	41 [20, 69]	41 [20, 69]	41 [22, 67]	41 [20, 69]	41 [20, 69]	41 [20, 69]
Min/maximum case	0/125	0/125	0/125	0/125	0/125	0/125
Low, n (%)	712 (24.2)	578 (24.6)	134 (22.8)	654 (23.6)	517 (23.3)	137 (24.7)
Intermediate, n (%)	1,046 (35.6)	822 (35.0)	224 (38.1)	1,015 (36.7)	821 (37.1)	194 (35.0)
Intermediate-to-high, n (%)	1,084 (36.9)	868 (37.0)	216 (36.7)	1,009 (36.4)	801 (36.2)	208 (37.5)
High, n (%)	95 (3.2)	81 (3.4)	14 (2.4)	91 (3.3)	76 (3.4)	15 (2.4)
Physician volume for secondary operator, median case	43 [22, 69]	43 [22, 69]	43 [22, 69]	43 [22, 69]	43 [22, 69]	43 [22, 69]
Min/maximum case	0/125	0/125	0/125	0/125	0/125	0/125
Low, n (%)	666 (22.7)	542 (23.1)	124 (21.1)	582 (21.0)	479 (21.6)	103 (18.6)
Intermediate, n (%)	988 (33.6)	781 (33.2)	207 (35.2)	949 (34.3)	757 (34.2)	192 (34.7)
Intermediate-to-high, n (%)	1,224 (41.7)	979 (41.7)	245 (41.7)	1,182 (42.7)	933 (42.1)	249 (44.9)
High, n (%)	59 (2.0)	47 (2.0)	12 (2.0)	56 (2.0)	46 (2.1)	10 (1.8)

COPD = chronic obstructive pulmonary disease; eGFR = estimated glomerular filtration rate; STS-PROM = Society of Thoracic Surgeons Predicted Risk of Mortality; TAVR = transcatheter aortic valve replacement.

Baseline antithrombotic therapy refers to patients who were on antithrombotic therapy prior to the TAVR procedure, regardless of its discontinuation at the time of the procedure.

### Diagnostic performance for predicting VARC-3 technical failure

**Figure 1:**
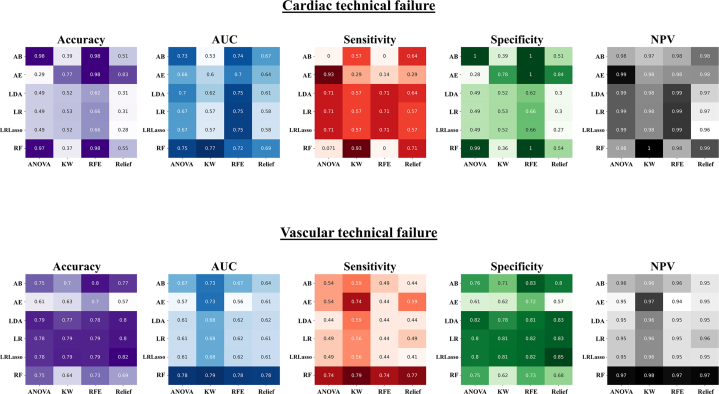
**Heat Maps of Model Performance** Heat maps illustrating accuracy, AUC, sensitivity, specificity, and negative predictive value for cardiac (*top*) and vascular technical failure (*bottom*) across different classifiers and feature selection methods. Classifiers include AdaBoost (AB), Autoencoder (AE), linear discriminant analysis (LDA), logistic regression (LR), Lasso logistic regression (LRLasso), and random forest (RF). Feature selection methods include analysis of variance (ANOVA), Kruskal-Wallis (KW), recursive feature elimination (RFE), and Relief. AUC = area under the curve; NPV = negative predictive value.

**Figure 2:**
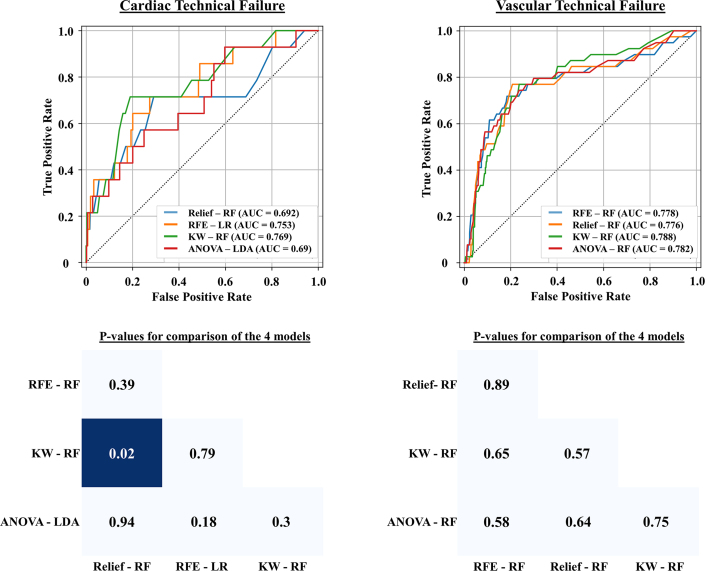
**Predictive Performance of Top 4 Models for VARC-3 Technical Failure** ROC curves for the top 4 models predicting VARC-3 technical failure (*top*) and statistical comparison of AUCs among the models (*bottom*). Dark blue indicates statistically significant differences (*P* < 0.05). TAVR = transcatheter aortic valve replacement; VARC = Valve Academic Research Consortium; other abbreviations as in Figure 1.

The diagnostic performance metrics for each machine learning model, combining different feature selection methods and classifiers, are summarized in Figure 1 and Table 2. For cardiac technical failure, the top-performing models included KW-RF (ROC-AUC: 0.769), RFE-LR (ROC-AUC: 0.753), Relief-RF (ROC-AUC: 0.692), and ANOVA-linear discriminant analysis (ROC-AUC: 0.690). For vascular technical failure, top models included KW-RF (ROC-AUC: 0.788), ANOVA-RF (ROC-AUC: 0.782), RFE-RF (ROC-AUC: 0.778), and Relief-RF (ROC-AUC: 0.776) (Figure 2). Although there were no statistically significant differences between most models, KW-RF showed significantly higher discrimination than Relief-RF for predicting cardiac technical failure and demonstrated the highest overall predictive performance for both cardiac and vascular technical failure (Figure 2, Central Illustration). The best-performing models demonstrate a negative predictive value of 0.995 for cardiac technical failure and 0.976 for vascular technical failure, respectively (Table 2, Figure 1, Central Illustration). Confusion matrixes, ROC curves, and statistical comparisons across all machine learning models are provided in Supplemental Figures 1 to 6.

**Table 2:** Performance Metrics of Machine-Learning Models for Predicting VARC-3 Technical Failure

Feature Selection	Classifier	AUC	Sensitivity	Specificity	NPV	Accuracy	F1 Score	Number of Cases			
								True Positive	True Negative	False Positive	False Negative
Cardiac technical failure											
Relief	AE	0.639	0.286	0.841	0.980	0.828	0.073	4	483	91	10
Relief	AB	0.671	0.643	0.507	0.983	0.510	0.059	9	291	283	5
Relief	LR	0.582	0.571	0.305	0.967	0.311	0.038	8	175	399	6
Relief	LRLasso	0.580	0.571	0.268	0.963	0.276	0.036	8	154	420	6
Relief	RF	0.692	0.714	0.544	0.987	0.548	0.070	10	312	262	4
Relief	LDA	0.614	0.643	0.298	0.972	0.306	0.042	9	171	403	5
RFE	AE	0.698	0.143	0.998	0.979	0.978	0.235	2	573	1	12
RFE	AB	0.739	0.000	1.000	0.976	0.976	0.000	0	574	0	14
RFE	LR	0.753	0.714	0.659	0.990	0.660	0.091	10	378	196	4
RFE	LRLasso	0.753	0.714	0.657	0.990	0.658	0.090	10	377	197	4
RFE	RF	0.722	0.000	1.000	0.976	0.976	0.000	0	574	0	14
RFE	LDA	0.749	0.714	0.617	0.989	0.619	0.082	10	354	220	4
KW	AE	0.603	0.286	0.777	0.978	0.765	0.055	4	446	128	10
KW	AB	0.534	0.571	0.389	0.974	0.393	0.043	8	223	351	6
KW	LR	0.567	0.571	0.528	0.981	0.529	0.055	8	303	271	6
KW	LRLasso	0.566	0.571	0.523	0.980	0.524	0.054	8	300	274	6
KW	RF	0.769	0.929	0.361	0.995	0.374	0.066	13	207	367	1
KW	LDA	0.618	0.571	0.523	0.980	0.524	0.054	8	300	274	6
ANOVA	AE	0.658	0.929	0.279	0.994	0.294	0.059	13	160	414	1
ANOVA	AB	0.729	0.000	1.000	0.976	0.976	0.000	0	574	0	14
ANOVA	LR	0.671	0.714	0.488	0.986	0.493	0.063	10	280	294	4
ANOVA	LRLasso	0.670	0.714	0.490	0.986	0.495	0.063	10	281	293	4
ANOVA	RF	0.752	0.071	0.993	0.978	0.971	0.105	1	570	4	13
ANOVA	LDA	0.698	0.714	0.490	0.986	0.495	0.063	10	281	293	4
Vascular technical failure											
Relief	AE	0.606	0.590	0.565	0.948	0.567	0.168	23	291	224	16
Relief	AB	0.635	0.436	0.800	0.949	0.774	0.259	17	412	103	22
Relief	LR	0.613	0.487	0.825	0.955	0.801	0.227	19	425	90	20
Relief	LRLasso	0.613	0.410	0.849	0.950	0.818	0.227	16	437	78	23
Relief	RF	0.776	0.769	0.682	0.975	0.688	0.283	30	351	164	9
Relief	LDA	0.616	0.436	0.825	0.951	0.798	0.221	17	425	90	22
RFE	AE	0.559	0.436	0.717	0.944	0.697	0.161	17	369	146	22
RFE	AB	0.672	0.487	0.827	0.955	0.803	0.214	19	426	89	20
RFE	LR	0.620	0.436	0.817	0.950	0.791	0.257	17	421	94	22
RFE	LRLasso	0.620	0.436	0.817	0.950	0.791	0.241	17	421	94	22
RFE	RF	0.778	0.744	0.734	0.974	0.735	0.258	29	378	137	10
RFE	LDA	0.624	0.436	0.810	0.950	0.783	0.233	17	417	98	22
KW	AE	0.732	0.744	0.619	0.970	0.628	0.220	29	319	196	10
KW	AB	0.727	0.590	0.709	0.958	0.700	0.217	23	365	150	16
KW	LR	0.677	0.564	0.808	0.961	0.791	0.275	22	416	99	17
KW	LRLasso	0.677	0.564	0.810	0.961	0.792	0.277	22	417	98	17
KW	RF	0.788	0.795	0.623	0.976	0.635	0.235	31	321	194	8
KW	LDA	0.676	0.590	0.779	0.962	0.765	0.261	23	401	114	16
ANOVA	AE	0.569	0.538	0.612	0.946	0.606	0.162	21	315	200	18
ANOVA	AB	0.672	0.538	0.761	0.956	0.745	0.230	21	392	123	18
ANOVA	LR	0.611	0.487	0.802	0.954	0.780	0.238	19	413	102	20
ANOVA	LRLasso	0.612	0.487	0.802	0.954	0.780	0.238	19	413	102	20
ANOVA	RF	0.782	0.744	0.748	0.975	0.747	0.293	29	385	130	10
ANOVA	LDA	0.614	0.436	0.819	0.950	0.792	0.228	17	422	93	22

Feature selection methods include analysis of variance (ANOVA), Kruskal-Wallis (KW), recursive feature elimination (RFE), and Relief.

Classifiers include AdaBoost (AB), autoencoder (AE), linear discriminant analysis (LDA), logistic regression (LR), Lasso logistic regression (LRLasso), and random forest (RF).

AUC = area under the curve; NPV = negative predictive value; VARC = Valve Academic Research Consortium.

### Predictors of VARC-3 technical failure

**Figure 3:**
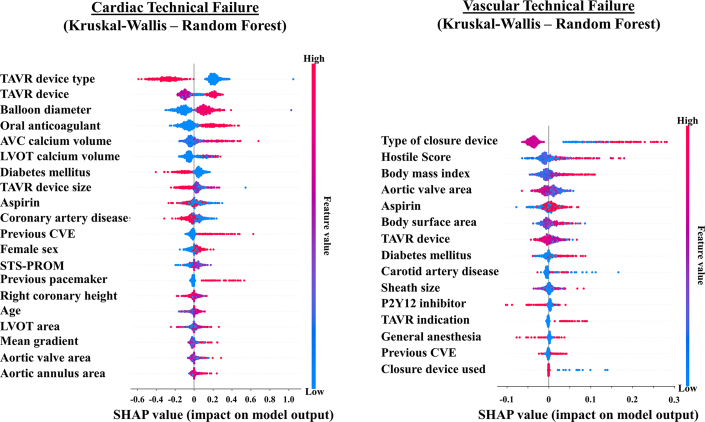
**SHAP Summary Plot for VARC-3 Technical Failure** SHAP summary plots illustrating the impact of each features on the best-performing models for cardiac (*left*) and vascular (*right*) technical failure. These plots visualize the contribution of each features to the predictive performance of each model. AVC = aortic valvular complex; CVE = cerebrovascular event; LVOT = left ventricular outflow tract; SHAP = SHapley Additive exPlanations; STS-PROM = Society of Thoracic Surgeons Predicted Risk of Mortality; other abbreviations as in Figure 1, Figure 2.

Figure 3 and Supplemental Figures 7 and 8 illustrate the SHAP summary plots of the top models. Across all models, procedural, CT-based anatomical, and baseline clinical features were consistently incorporated as predictors of VARC-3 technical failure. Specifically, for cardiac technical failure, the consistently identified predictors included TAVR device size, size of predilatation balloon, aortic valvular complex calcium volume, age, diabetes, previous cerebrovascular events, previous pacemaker implantation, and use of oral anticoagulants. For vascular technical failure, the top models consistently identified the type of vascular closure device, TAVR device, sheath size, hostile score, aortic valve area, body mass index, body surface area, diabetes, previous cerebrovascular events, use of aspirin, use of a P2Y12 inhibitor, and TAVR indication (native TAVR vs valve-in-valve TAVR) as predictive features.

## Discussion

In this analysis of a large prospective registry cohort of 2,937 eligible patients, we describe machine learning algorithms for a risk prediction model of TAVR technical failure. TAVR has evolved as an alternative to surgical aortic valve replacement and has rapidly expanded due to the improved safety profile and favorable clinical outcomes reported in the randomized controlled trials.^1^^,^^2^^,^^29^ However, even in a contemporary TAVR population, there remains a non-negligible rate of procedural complications in the real-world setting. In the multicenter OPERA-TAVI registry, up to 7% of patients undergoing TAVR with newer-generation devices (SAPIEN 3 Ultra or Evolut PRO) developed VARC-3 technical failure.^8^ Technical failure is a procedural complication requiring rescue interventions for stabilization. However, the occurrence of technical failure is associated with an increased risk of adverse events even after the rescue procedures. In a previous study of 1,624 patients undergoing TAVR, technical failure was associated with a two-fold increased risk of cardiovascular death and stroke, and the risk was consistent between the type of technical failure (cardiac or vascular).^4^ Indeed, although the rate of bailout surgical intervention for the treatment of life-threatening complications has declined to <1% in contemporary TAVR populations, short-term mortality after rescue procedures remains considerable even in low-risk patients.^5^^,^^30^ Therefore, accurately predicting the risk of procedural failure during preprocedural planning is crucial for optimal patient selection.

In the present study, we leveraged a comprehensive data set with multimodal clinical, electrocardiographic, and invasive hemodynamics, imaging, and procedural features to better predict technical failure of TAVR. Our best models show moderate-to-high discrimination for both cardiac and vascular technical failure. Notably, our models consistently demonstrated excellent negative predictive value, suggesting a low likelihood of incorrectly referring unsuitable patients for TAVR. Recently, several AI-based predictive models for TAVR outcomes have been developed.^10^, ^11^, ^12^ The TAVI risk machine (TRIM) score, which was trained and cross-validated on data from 22,283 patients within the nationwide GARY registry and validated on data from 6,693 patients within the nationwide SwissTAVI registry, is a machine learning-based model for predicting 30-day survival after TAVR. The model outperformed STS-PROM in both the derivation (TRIM: C-statistic 0.79; 95% CI: 0.74-0.83; STS-PROM: C-statistic 0.69; 95% CI: 0.65-0.74) and validation cohorts (TRIM: C-statistic 0.75; 95% CI: 0.72-0.79; STS-PROM: C-statistic 0.67; 95% CI: 0.63-0.70).^11^ The integration of AI-based decision-making is expected to increase across all phases of patient care for patients with cardiovascular disease.^31^ To our knowledge, this is the first machine learning-based model specifically designed to predict TAVR procedural failure using VARC-3 definitions, integrating multimodal clinical, electrocardiographic, and invasive hemodynamics, imaging, and procedural data. Integrating procedural failure risk prediction into preprocedural planning may help refine patient selection, optimize procedural strategies, and improve overall outcomes.

In the present analysis, we identified the key contributing features to the development of VARC-3 technical failure using SHAP analysis. Consistent with previous studies, we observed that baseline clinical characteristics, CT-based anatomical features, and procedural factors were associated with procedural failure. For instance, a severely calcified device landing zone is a crucial anatomical consideration, as it increases the risk of annular rupture and coronary obstruction.^15^^,^^32^ Similarly, the severity and extent of iliofemoral atherosclerosis are associated with a higher risk of vascular complications.^7^ Several baseline patient characteristics, such as diabetes mellitus and previous cerebrovascular events, may reflect an advanced atherosclerotic burden.^33^^,^^34^ In the present study, preprocedural antithrombotic management emerged as an important factor influencing procedural failure. The POPular PAUSE TAVI randomized trial (n = 869) demonstrated that patients who continued oral anticoagulants had a higher incidence of procedural-related bleeding compared with those who interrupted anticoagulation before TAVR (28.3% vs 19.2%; risk difference 9.1%, 95% CI: 3.4-14.8).^35^ These findings reinforce the need for optimized preprocedural antithrombotic strategies, particularly in patients at high risk of vascular complications. Similarly, procedural factors play an important role in the development of procedural complications. In a single-center analysis of 1,638 patients undergoing TAVR, suboptimal transcatheter heart valve sizing was associated with an increased risk of valve migration, embolization, and bailout second valve implantation, leading to a lower device success rate.^36^ The improved profile of newer-generation suture-based vascular closure devices over first-generation suture-based and plug-based devices has also been demonstrated in previous studies.^9^^,^^37^ Notably, across all proposed models, baseline clinical, CT-based anatomical, and procedural features were consistently identified as the key predictors of VARC-3 technical failure. These findings highlight the importance of integrating multimodal data to enhance risk stratification and optimize procedural planning in TAVR.

Although the number of centers performing TAVR is consistently increasing, the increase may lead to a dilution of the quality of patient selection and procedural quality.^38^ For patients at high risk of technical failure, the Heart Team must carefully reassess the treatment strategy, considering alternative options such as conservative management or surgical aortic valve replacement. Similarly, high-risk cases should be prioritized for treatment at experienced TAVR centers equipped with comprehensive bailout options to address potential complications.^6^ Machine learning-based models may be beneficial in guiding the future expansion of TAVR, refining patient selection, optimizing procedural strategies, and ensuring that high-risk cases receive care at appropriately equipped centers.

### Study limitations

The findings of our study should be interpreted in light of several limitations. First, although the study was based on a large prospective TAVR registry with findings reinforced by detailed multimodal information, including clinical, electrocardiographic and invasive hemodynamics, and imaging data and granular procedural outcomes, external validation was not performed due to the lack of an independent data set. Furthermore, the number of technical failure events was modest, which constrains statistical power and the precision of performance estimates for this outcome. This limitation is inherent to rare-event prediction and should be considered when interpreting the cardiac model, which we regard as exploratory. Nevertheless, our modeling strategy, which incorporates dimensionality reduction, stratified training/testing, and rigorous model development and evaluation, was specifically designed to mitigate overfitting and optimize performance despite low event rates. Moreover, several key features for the development of technical failure, such as ultrasound-guided puncture, were not systematically captured in the database and could not be incorporated into the model development. However, we conducted a standardized evaluation, ensuring that each step of the machine learning development and evaluation process was standardized for consistency and reproducibility. To facilitate further evaluation, we have made the model openly accessible, allowing other researchers to validate its performance using their own data sets. Second, the results of the present study reflect the experience of a single high-volume center with high-experience operators and may not be generalizable to other TAVR centers. Notably, in the present analysis, operator experience suggested as a key feature for procedural safety was not selected as a key feature, regardless of the type of methodology and technical failure. External validation in independent TAVR cohorts will be essential to further refine and improve the predictive performance of these models. Finally, although we excluded patients treated with early-generation devices no longer in clinical use, some of the included devices represent predecessors of their current-generation versions. However, these platforms share fundamental procedural techniques and valve design principles. Moreover, device uptake varies across institutions, and even predecessor platforms remain in use in some centers. Including a diverse population treated with structurally similar devices may improve model generalizability and robustness, and may be preferable to limiting the model strictly to the latest technology. Future refinements should aim to validate and adapt the model across centers with varying device portfolios and practice patterns.

## Conclusions

In the present study, we developed machine learning algorithms for predicting VARC-3 technical failure in patients with severe aortic stenosis undergoing TAVR. Our best-performing models demonstrated moderate-to-high discrimination for both cardiac and vascular technical failure, with excellent negative predictive value, ensuring a low likelihood of misclassifying suitable TAVR candidates. Across all proposed models, baseline clinical characteristics, CT-based anatomical features, and procedural variables were consistently identified as the key determinants of VARC-3 technical failure, underscoring the importance of integrating multimodal data into preprocedural risk assessment.

Perspectives **COMPETENCY IN MEDICAL KNOWLEDGE:** VARC-3 technical failure is associated with unfavorable clinical outcomes. However, its prediction remains challenging due to the complex interplay of clinical, anatomical, and procedural factors. In this study, we developed and validated machine learning models to predict VARC-3 technical failure in patients undergoing TAVR. Our best-performing models demonstrated moderate-to-high predictive accuracy for both cardiac and vascular technical failure, with excellent negative predictive value, ensuring a low likelihood of misclassifying suitable TAVR candidates.

**TRANSLATIONAL OUTLOOK:** External validation in independent TAVR cohorts will be essential to further refine and improve the predictive performance of these models.

## Funding support and author disclosures

Dr Shiri has received travel expenses from Alnylam and Bayer, and speaker fees and consultancy fees to the institution from Pfizer. Dr Samim has received funding for an online course from Edwards Lifesciences. Dr Bartkowiak has received grants from 10.13039/100008273Novartis Foundation. Dr Praz has received travel expenses from Abbott, Edwards Lifesciences, and Polares Medical. Dr Lanz reports speaker fees to the institution from Edwards Lifesciences and Abbott and served as advisory board member for Abbott. Dr Stortecky has received institutional research grants from 10.13039/100006520Edwards Lifesciences, 10.13039/100004374Medtronic, Abbott Vascular, and 10.13039/100008497Boston Scientific; and has received speaker fees from Boston Scientific. Dr Reineke reports travel expenses from Abbott, Edwards Lifesciences and Medtronic and has proctor and consulting contracts with Abbott and Medtronic. Dr Windecker reports research, travel, and/or educational grants to the institution from 10.13039/100000046Abbott, 10.13039/100020297Abiomed, 10.13039/100006400Alnylam, 10.13039/100015362Amicus Therapeutics, 10.13039/100002429Amgen, Astra Zeneca, Bayer, B.Braun, Bioanalytica, Biotronik, Boehringer Ingelheim, 10.13039/100008497Boston Scientific, Bristol Myers Squibb, Cordis Medical, CorFlow Therapeutics, CSL Behring, Daiichi Sankyo, 10.13039/100006520Edwards Lifesciences, Fumedica, GE Healthcare, 10.13039/100020333Guerbet, IACULIS, 10.13039/100022880Inari Medical, Janssen AI, Johnson & Johnson, 10.13039/501100023518Medalliance, 10.13039/100004374Medtronic, MSD Merck Sharp & Dohme, Neovii Pharmaceutica, Neutromedics AG, 10.13039/100004336Novartis, 10.13039/501100004191Novo Nordisk, 10.13039/501100024065OM Pharma, Optimapharm, Orchestra BioMed, 10.13039/100004319Pfizer, Philips AG, Sanofi-Aventis, Servier, Shockwave Medical, Siemens Healthcare, Sinomed, SMT Sahajanand Medical Technologies, Vascular Medical, and V-Wave; he serves as advisory board member and/or member of the steering/executive group of trials funded by Abbott, Amgen, Abiomed, Edwards Lifesciences, EnCarda Inc, Medtronic, Novartis, and Sinomed with payments to the institution but no personal payments; and is also a member of the steering/executive committee group of several investigator-initiated trials that receive funding by industry without impact on his personal remuneration. Dr Pilgrim reports research grants from the Swiss National Science Foundation, the Swiss Heart Foundation, the Swiss Polar Institute, the Bangerter-Rhyner Foundation, the Mach-Gaensslen Foundation, and the Monsol Foundation; research, travel, or educational grants to the institution without personal remuneration from Biotronik, Boston Scientific, Edwards Lifesciences, and ATSens; speaker fees and consultancy fees to the institution from Biotronik, Boston Scientific, Edwards Lifesciences, Abbott, Medtronic, Biosensors, and Highlife. Dr Gräni received research funding from the GAMBIT foundation for this work; he received funding from the Swiss National Science Foundation, InnoSuisse, Center for Artificial Intelligence in Medicine University Bern, Novartis Foundation for Medical-Biological Research, Swiss Heart Foundation, Schmieder-Bohrisch Foundation, Gottfried, and Julia Bangerter-Rhyner foundation, outside of the submitted work; furthermore, funding to the institution was received from Alnylam Pharmaceuticals, AstraZeneca, Pfizer, Bayer, outside of the submitted work and without impact on his personal remuneration. All other authors have reported that they have no relationships relevant to the contents of this paper to disclose.
