# Supplementary material for: Multimodal Machine Learning-Based Technical Failure Prediction in Patients Undergoing Transcatheter Aortic Valve Replacement
Source: JACC Adv. 2025 Sep 18;4(10):102168. doi: 10.1016/j.jacadv.2025.102168 (PMC12481615; doi:10.1016/j.jacadv.2025.102168)

**SUPPLEMENTAL APPENDIX**

##

## **Supplemental Information. General definition of all features**

**Clinical variables**: indication for TAVR (native aortic stenosis/severe stenosis of an aortic bioprosthesis/severe regurgitation of an aortic bioprosthesis/degenerative transcatheter heart valve prosthesis), urgent procedure (yes/no), sex (male/female), age (years), height (cm), weight (kg), body mass index (kg/m^2^), body surface area (m^2^), logistic EuroScore (%), Linear EuroScore (%), STS-PROM (%), dyspnea (yes/no), NYHA functional class, NYHA functional class III or IV (yes/no), syncope (yes/no), stable angina pectoris (yes/no), CCS Angina Class (I/II/III/IV), CCS Angina Class III or IV (yes/no), diabetes mellitus (yes/no), diabetes mellitus – diet therapy (yes/no), diabetes mellitus – oral medication (yes/no), diabetes mellitus - insulin (yes/no), hypertension (yes/no), dyslipidemia (yes/no), renal failure (eGFR <60 mL/min/1.73 m^2^) (yes/no), dialysis (yes/no), chronic obstructive pulmonary disease (yes/no), history of cerebrovascular event (yes/no), previous pacemaker implantation (yes/no), previous defibrillator implantation (yes/no), previous aortic valvuloplasty (yes/no), coronary artery disease (yes/no), number of diseased vessels involved (1/2/3), history of PCI (yes/no), date of last PCI (date), type of PCI (drug-eluting stent/bare-metal stent/plain balloon angioplasty), history of myocardial infarction (yes/no), peripheral artery disease (yes/no), history of cardiac surgery (yes/no), number of previous cardiac surgery (number), date of previous cardiac surgery (date), carotid artery disease (yes/no), atrial fibrillation (yes/no), type of atrial fibrillation (paroxymal/persistent/permanent), CHADS2 Score, Chads2Vasc2 Score, HAS Bled Score, aspirin (yes/no), P2Y12 antagonist (yes/no), clopidogrel (yes/no), prasugrel (yes/no), ticagrelor (yes/no), oral anticoagulant (yes/no), dabigatran (yes/no), apixaban (yes/no), rivaroxaban (yes/no), edoxaban (yes/no), statin (yes/no), angiotensin-converting enzyme inhibitor (yes/no), angiotensin II receptor blockers (yes/no), beta-blocker (yes/no), calcium channel blocker (yes/no), diuretics (yes/no), steroids (yes/no), immunosuppressive drugs (yes/no), sodium-glucose cotransporter-2 inhibitor (yes/no), angiotensin receptor-neprilysin inhibitors (yes/no);

**Laboratory markers**: troponin type (T/I/hs), troponin level (ng/l), creatinine (µmol/l), eGFR (mL/min/1.73 m^2^), hemoglobin (g/l), thrombocytes (10^9^/l), creatine kinase (U/l), creatine kinase-myocardial band (U/l), brain natriuretic peptide (pg/ml), albumin (g/l), leucocytes (g/l);

**Electrocardiogram**: rhythm (sinus/atrial fibrillation/atrial flutter/paced rhythm/other), heart rate (bpm), atrio-ventricular block (yes/no), degree of AVb (1st/2nd/3rd), intraventricular conduction delay (none/RBBB/LBBB);

**Computed tomography**: type of valve (tricuspid/bicuspid/valve-in-valve), aortic annulus maximum diameter (mm), aortic annulus minimum diameter (mm), aortic annulus mean diameter (mm), aortic annulus area (mm^2^), aortic annulus area-derived diameter (mm), aortic annulus perimeter (mm), aortic annulus perimeter-derived diameter (mm), aortic annulus eccentricity, left coronary height (mm), right coronary height (mm), ascending aorta diameter (mm), sinotubular junction (mm), sinus of Valsalva (mm), LVOT dimeter (mm), LVOT area (mm^2^), aortic angulation (˚), membranous septum length (mm), total aortic valvular complex calcium volume (mm^3^), aortic valvular complex calcium volume (RCC) (mm^3^), aortic valvular complex calcium volume (LCC) (mm^3^), aortic valvular complex calcium volume (NCC) (mm^3^), total LVOT calcium volume (mm^3^), LVOT calcium volume (RCC) (mm^3^), LVOT calcium volume (LCC) (mm^3^), LVOT calcium volume (NCC) (mm^3^), severity of LVOT calcification (none/mild/moderate/severe), total device landing zone calcium volume (mm^3^), device landing zone calcium volume (RCC) (mm^3^), device landing zone calcium volume (LCC) (mm^3^), device landing zone calcium volume (NCC) (mm^3^), porcelain aorta (yes/no), Hostile score; number of segments with significant disease (number), Hostile score; iliac artery disease involving the aortic bifurcation (yes/no), Hostile score; total diseased lesion length >100 mm (yes/no), Hostile score; presence of ≥180° arch calcified lesion (yes/no), Hostile score; minimum lumen diameter < 5mm (yes/no), Hostile score; lesion in tortuous segment (yes/no), Hostile score; presence of total obstruction (yes/no), Hostile score (number);

**Transthoracic echocardiography**: aortic valve area (cm^2^), indexed aortic valve area (cm^2^), mean gradient (mmHg), peak gradient (mmHg), left ventricular ejection fraction (%), aortic regurgitation (none/mild/moderate/severe), mitral regurgitation (none/mild/moderate/severe), tricuspid regurgitation (none/mild/moderate/severe), right ventricular dysfunction (yes/no), severe pulmonary hypertension (yes/no), left ventricular mass (g), left ventricular mass index (g/m^2^), left ventricular end-diastolic diameter (mm), left ventricular end-systolic diameter (mm), aortic valve annulus diameter (mm), aortic sinus diameter (mm), sinotubular junction diameter (mm), ascending aorta diameter (mm);

**Transesophageal echocardiography**: aortic valve area (cm^2^), indexed aortic valve area (cm^2^), mean gradient (mmHg), peak gradient (mmHg), left ventricular ejection fraction (%), aortic regurgitation (none/mild/moderate/severe), mitral regurgitation (none/mild/moderate/severe), tricuspid regurgitation (none/mild/moderate/severe), aortic valve annulus diameter (mm), aortic sinus diameter (mm), sinotubular junction diameter (mm), ascending aorta diameter (mm);

**Invasive measurement**: aortic valve area (cm^2^), indexed aortic valve area (cm^2^), mean gradient (mmHg), peak gradient (mmHg), left ventricular ejection fraction (%), aortic regurgitation (none/mild/moderate/severe);

**Procedural features**: type of anesthesia (local/general), main access site (femoral/transapical/subclavian/other), type of access (percutaneous/surgical), use of cerebral protection device (yes/no), type of cerebral protection device, balloon diameter for pre-dilatation (mm), use of vascular closure device (yes/no), vascular closure device (PerClose or ProGlide/Prostar/MANTA), TAVR device (SAPIEN 3/SAPIEN 3 Ultra/Evolut R/Evolut PRO/Evolut PRO Plus/Acurate Neo/Acurate Neo 2/Portico/Navitor), TAVR device type (balloon-expandable valve/self-expanding valve), TAVR device size (mm), sheath size (Fr), operator experience.

CCS = Canadian Cardiovascular Society; eGFR = estimated glomerular filtration rate; LCC = left coronary cusp; LVOT = left ventricular outflow tract; NCC = non-coronary cusp; NYHA = Yew York Heart Association; PCI = percutaneous coronary intervention; RCC = right coronary cusp; STS-PROM = Society of Thoracic Surgeons Predicted Risk of Mortality; TAVR = transcatheter aortic valve replacement.

## **Supplemental Table 1. Procedural results in Train/Validation and Hold out test sets**

|  | **Cardiac technical failure** | | | **Vascular technical failure** | | |
| --- | --- | --- | --- | --- | --- | --- |
|  | **Overall**  **N = 2,937** | **Train/Validation set**  **N = 2,349** | **Hold-out Test Set**  **N = 588** | **Overall**  **N = 2,769** | **Train/Validation set**  **N = 2,215** | **Hold-out Test Set**  **N = 554** |
| **Cardiac technical failure, n (%)** | 70 (2.4) | 56 (2.4) | 14 (2.4) | 65 (2.3) | 55 (2.5) | 10 (1.8) |
| Valve dislocation/embolization, n (%) | 23 (0.8) | 17 (0.7) | 6 (1.0) | 23 (0.8) | 18 (0.8) | 5 (0.9) |
| Reposition with snare, n (%) | 3 (0.1) | 2 (0.1) | 1 (0.2) | 2 (0.1) | 2 (0.1) | 0 (0.0) |
| Second valve implantation, n (%) | 24 (0.8) | 15 (0.6) | 9 (1.5) | 23 (0.8) | 18 (0.8) | 5 (0.9) |
| Valve retrieval, n (%) | 23 (0.8) | 20 (0.9) | 3 (0.5) | 21 (0.8) | 19 (0.9) | 2 (0.4) |
| Annular rupture, n (%) | 25 (0.9) | 23 (1.0) | 2 (0.3) | 24 (0.9) | 19 (0.9) | 5 (0.9) |
| Coronary artery obstruction, n (%) | 11 (0.4) | 9 (0.4) | 2 (0.3) | 11 (0.4) | 8 (0.4) | 3 (0.5) |
| Cardiac tamponade, n (%) | 15 (0.5) | 13 (0.6) | 2 (0.3) | 15 (0.5) | 14 (0.6) | 1 (0.2) |
| Unplanned cardiac intervention, n (%) | 36 (1.2) | 30 (1.3) | 6 (1.0) | 35 (1.3) | 29 (1.3) | 6 (1.1) |
| Conversion to surgery, n (%) | 17 (0.6) | 16 (0.7) | 1 (0.2) | 16 (0.6) | 16 (0.7) | 0 (0.0) |
| **Vascular technical failure, n (%)** | 198 (6.7) | 157 (6.7) | 41 (7.0) | 195 (7.0) | 156 (7.0) | 39 (7.0) |
| Stent placement for access-related complication, n (%) | 187 (6.4) | 150 (6.4) | 37 (6.3) | 186 (6.7) | 150 (6.8) | 36 (6.5) |
| Vascular surgery for access-related complication, n (%) | 20 (0.7) | 15 (0.6) | 5 (0.9) | 17 (0.6) | 12 (0.5) | 5 (0.9) |

## **Supplemental Figure 1. Confusion matrix for VARC-3 cardiac technical failure prediction in holdout test set**


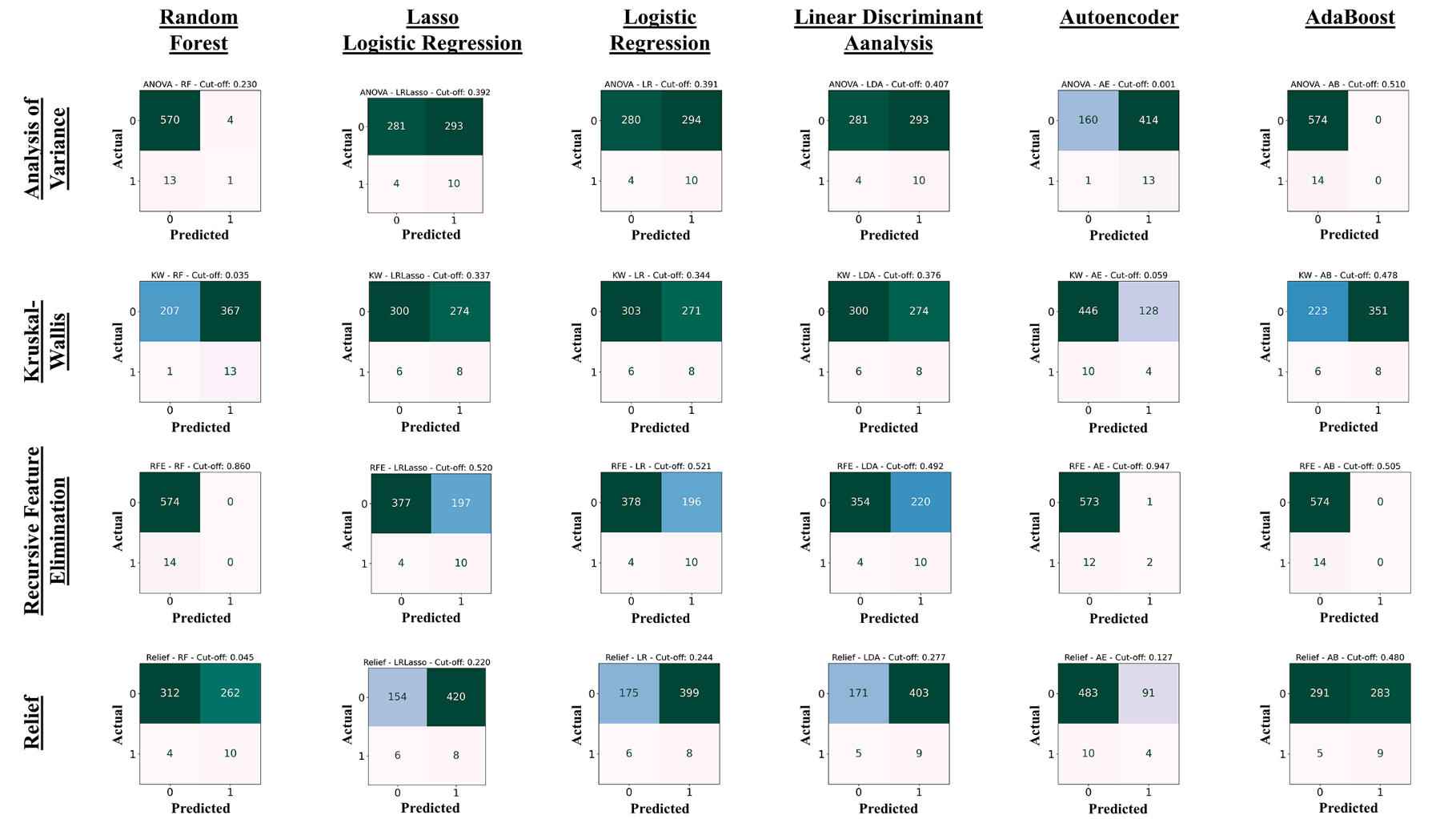


## **Supplemental Figure 2.** **Confusion matrix for VARC-3 vascular technical failure prediction in holdout test set**


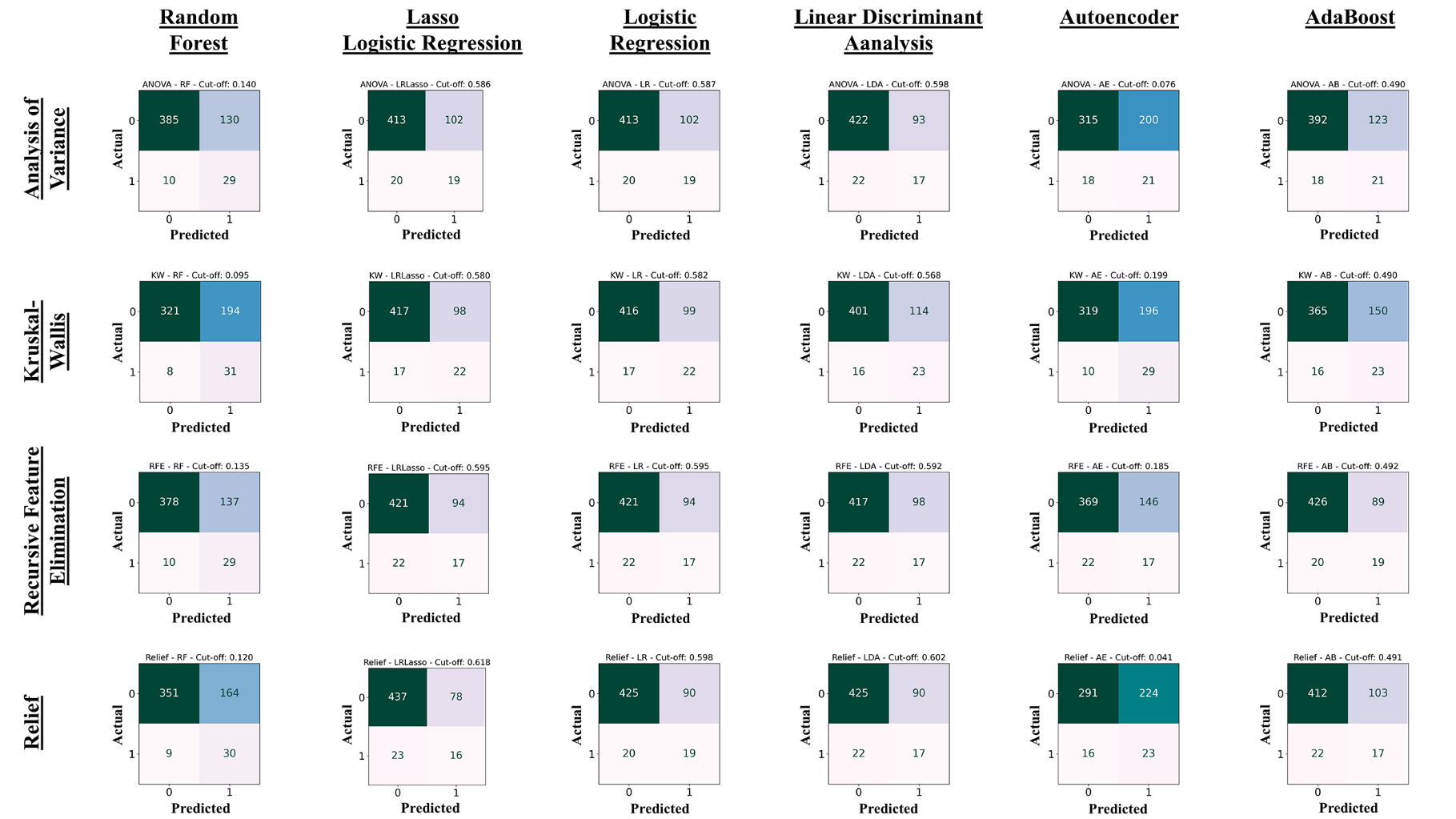


## **Supplemental Figure 3.** **Predictive value of each model on VARC-3 cardiac technical failure**


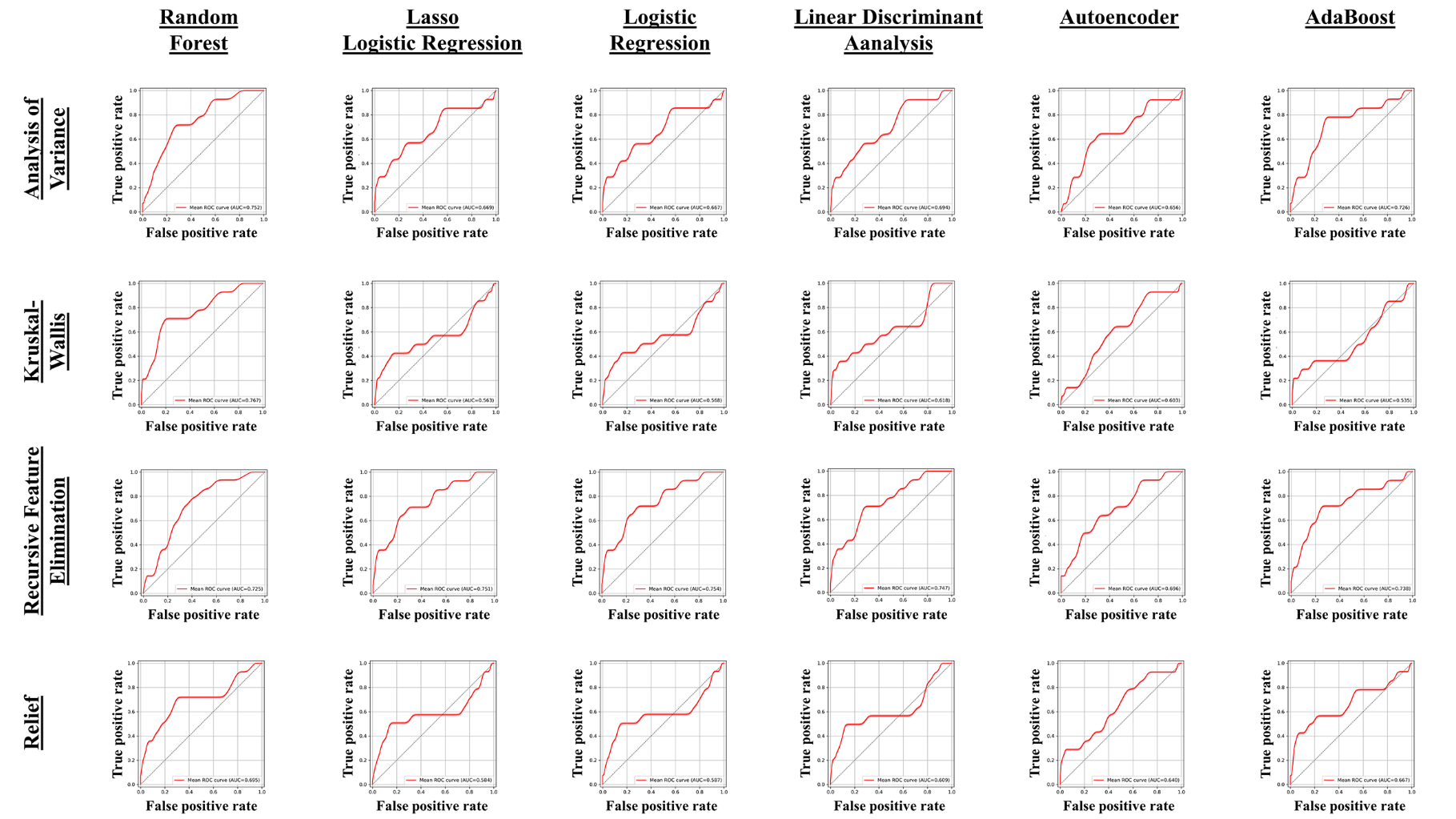


## **Supplemental Figure 4.** **Predictive value of each model on VARC-3 vascular technical failure**


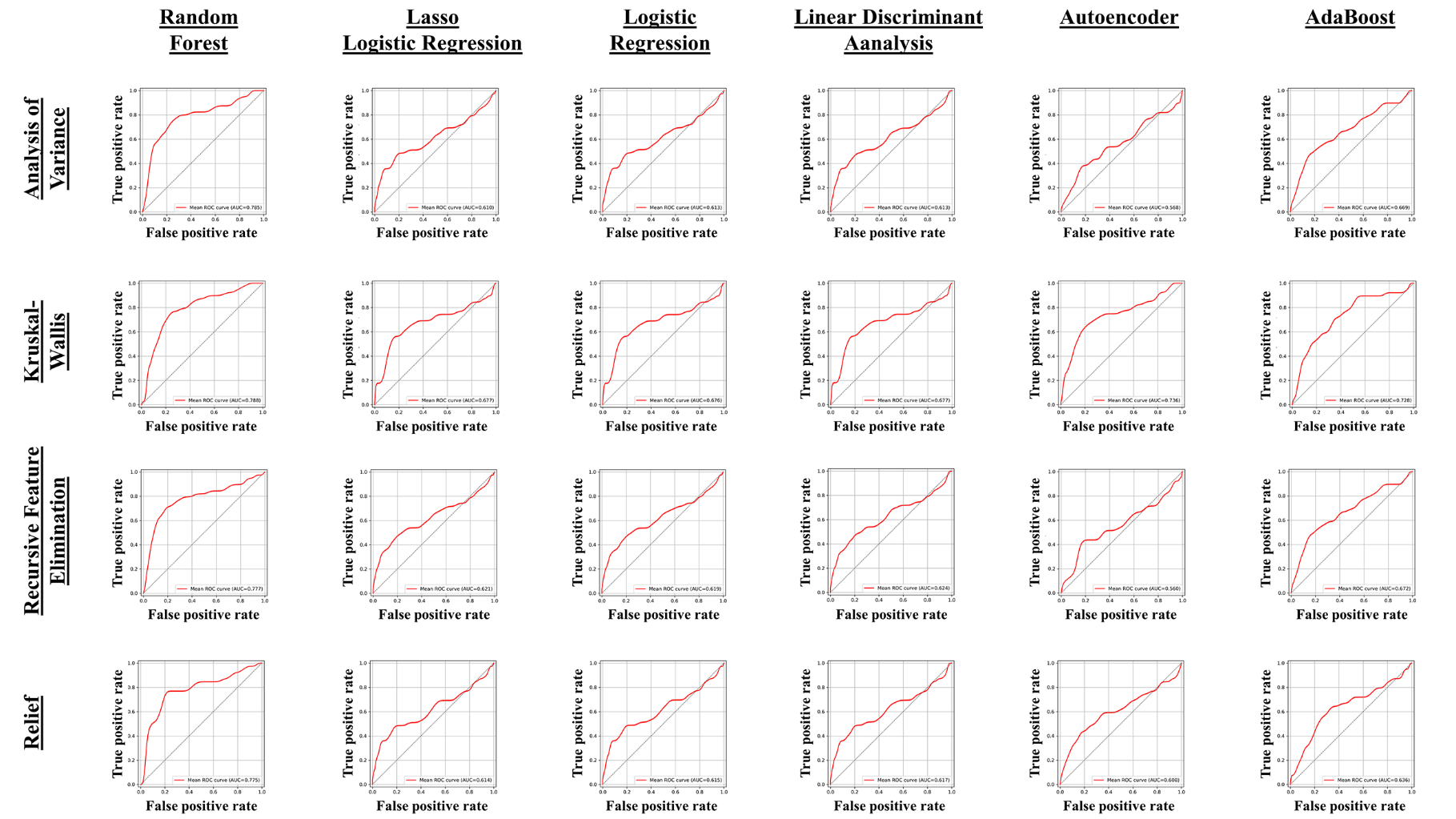


## **Supplemental Figure 5.** **Statistical comparison of different predictive models on VARC-3 cardiac technical failure**

Dark blue indicates statistically significant (P <0.05).


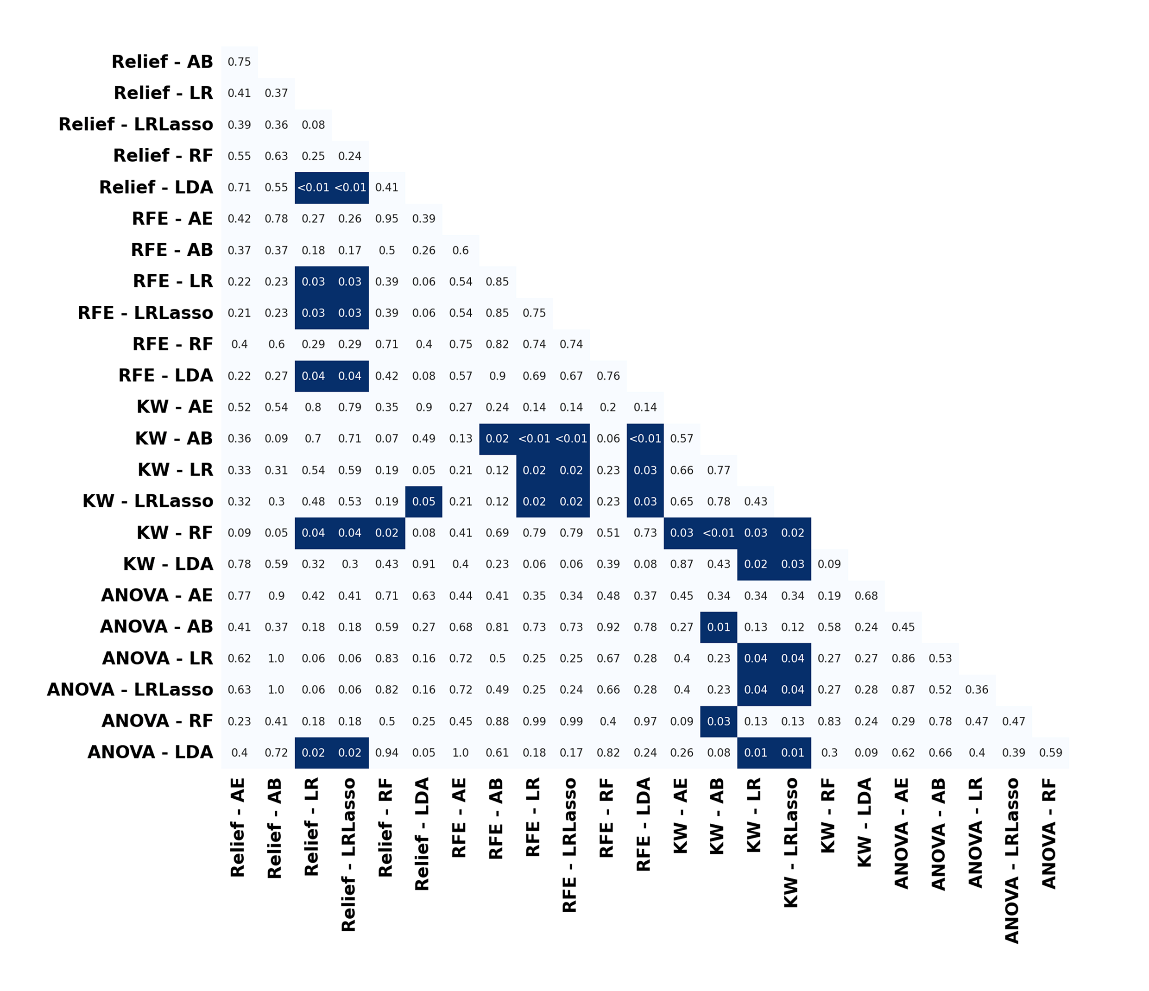


## **Supplemental Figure 6.** **Statistical comparison of different predictive models on VARC-3 vascular technical failure**

Dark blue indicates statistically significant (P <0.05).


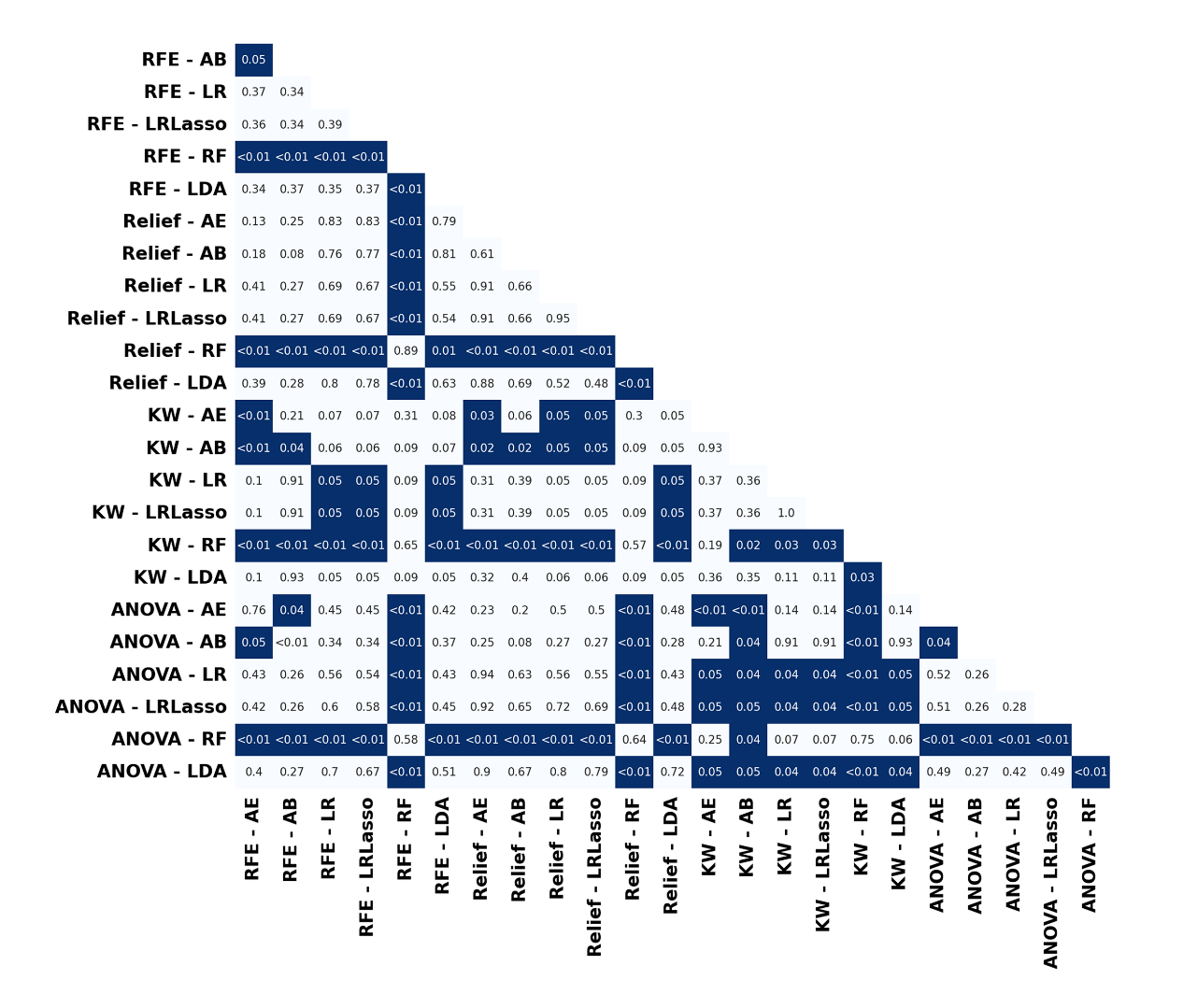


## **Supplemental Figure 7.** **SHAP summary plot for VARC-3 cardiac technical failure**

SHAP summary plots showing the impact of various features on the prediction models for VARC-3 cardiac technical failure.

This visualization highlights the contribution of individual features to the predictive performance of each model for predicting cardiac technical failure.


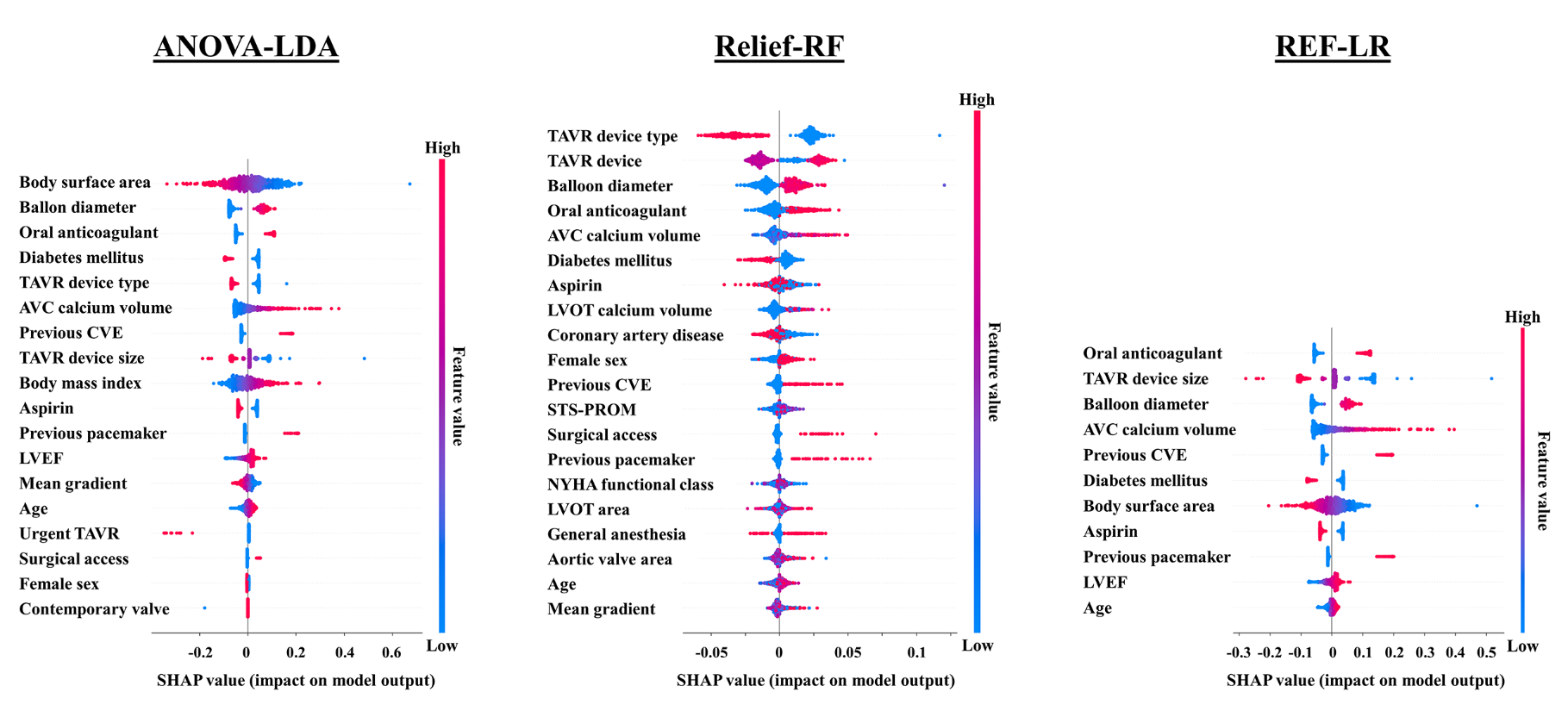


## **Supplemental Figure 8.** **SHAP summary plot for VARC-3 technical failure**

SHAP summary plots showing the impact of various features on the prediction models for VARC-3 vascular technical failure.

This visualization highlights the contribution of individual features to the predictive performance of each model for predicting vascular technical failure.


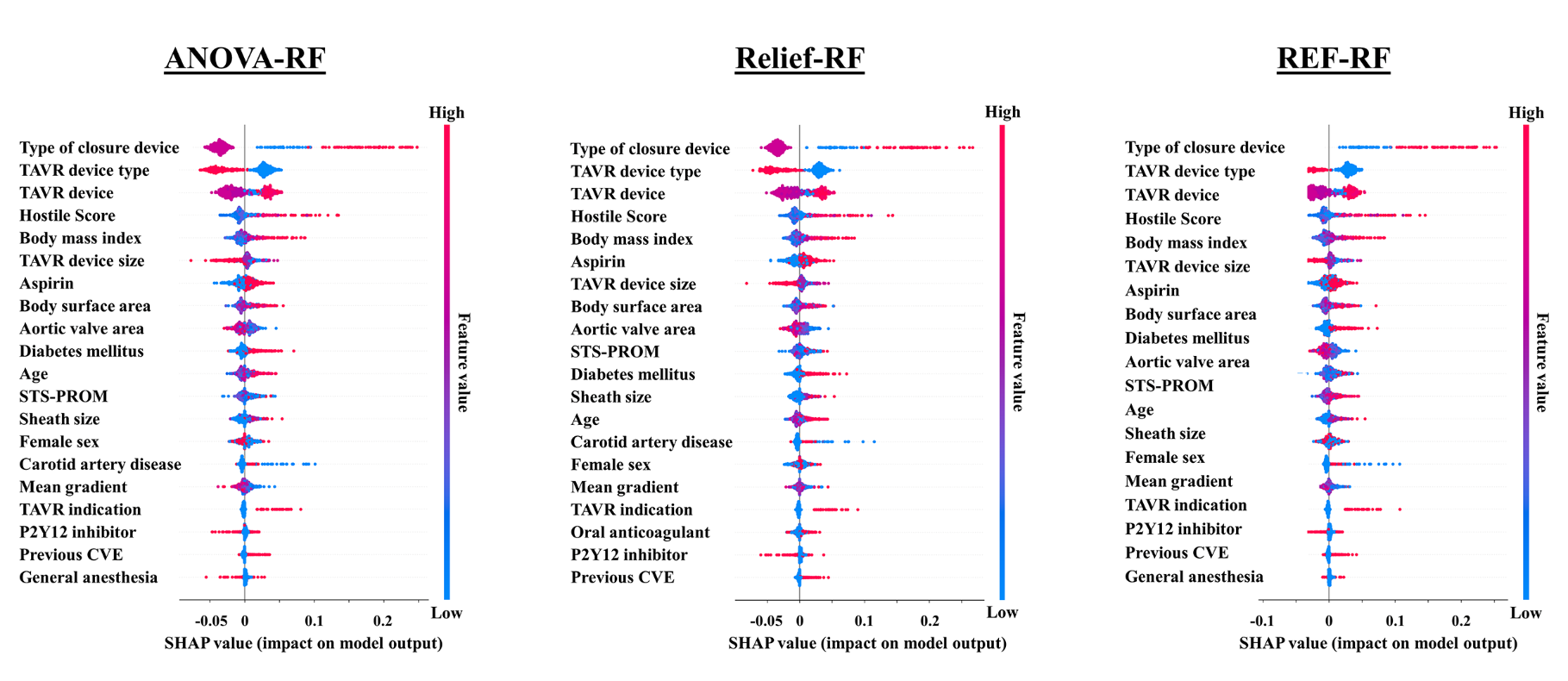

Supplement: Supplemental Data [file mmc1.docx]
